# Influenza Vaccine Effectiveness against Hospitalisation with Confirmed Influenza in the 2010–11 Seasons: A Test-negative Observational Study

**DOI:** 10.1371/journal.pone.0068760

**Published:** 2013-07-16

**Authors:** Allen C. Cheng, Mark Holmes, Louis B. Irving, Simon G. A. Brown, Grant W. Waterer, Tony M. Korman, N. Deborah Friedman, Sanjaya Senanayake, Dominic E. Dwyer, Stephen Brady, Grahame Simpson, Richard Wood-Baker, John Upham, David Paterson, Christine Jenkins, Peter Wark, Paul M. Kelly, Tom Kotsimbos

**Affiliations:** 1 Infectious Diseases Unit, Alfred Hospital, Melbourne, Victoria, Australia; 2 Department of Epidemiology and Preventive Medicine, Monash University, Melbourne, Victoria, Australia; 3 Department of Respiratory and Sleep Medicine, Royal Adelaide Hospital, Adelaide, South Australia, Australia; 4 Department of Medicine, The University of Adelaide, Adelaide, South Australia, Australia; 5 Department of Emergency Medicine, Royal Perth Hospital, and the Centre for Clinical Research in Emergency Medicine, Western Australian Institute for Medical Research, University of Western Australia, Perth, Western Australia, Australia; 6 Department of Respiratory Medicine, Royal Perth Hospital, School of Medicine and Pharmacology, University of Western Australia, Perth, Western Australia, Australia; 7 Infectious Diseases and Microbiology, Monash Medical Centre, Melbourne, Victoria, Australia; 8 Monash University, Melbourne, Victoria, Australia; 9 The Geelong Hospital, Barwon Health, Geelong, Victoria, Australia; 10 Infectious Diseases Unit, The Canberra Hospital, Canberra, Australian Capital Territory, Australia; 11 Medical School, Australian National University, Canberra, Australian Capital Territory, Australia; 12 Centre for Infectious Diseases and Microbiology Laboratory Services, Westmead Hospital, University of Sydney, Sydney, New South Wales, Australia; 13 Alice Springs Hospital, Alice Springs, Northern Territory, Australia; 14 Cairns Base Hospital, Cairns, Queensland, Australia; 15 Menzies Research Institute, University of Tasmania, Hobart, Tasmania, Australia; 16 Princess Alexandra Hospital, The University of Queensland Brisbane, Queensland, Australia; 17 Royal Brisbane and Women’s Hospital, The University of Queensland, Brisbane, Queensland, Australia; 18 Concord Hospital, University of Sydney, Woolcock Institute of Medical Research, Sydney, New South Wales, Australia; 19 University of Newcastle and John Hunter Hospital, Newcastle, New South Wales, Australia; 20 Population Health Division, ACT Government Health Directorate, Canberra, Australian Capital Territory, Australia; 21 Medical School, Australian National University, Canberra, Australian Capital Territory, Australia; 22 Allergy, Immunology and Respiratory Medicine Unit, Alfred Hospital, Department of Medicine, Monash University, Melbourne, Victoria, Australia; National Institutes of Health, United States of America

## Abstract

Immunisation programs are designed to reduce serious morbidity and mortality from influenza, but most evidence supporting the effectiveness of this intervention has focused on disease in the community or in primary care settings. We aimed to examine the effectiveness of influenza vaccination against hospitalisation with confirmed influenza. We compared influenza vaccination status in patients hospitalised with PCR-confirmed influenza with patients hospitalised with influenza-negative respiratory infections in an Australian sentinel surveillance system. Vaccine effectiveness was estimated from the odds ratio of vaccination in cases and controls. We performed both simple multivariate regression and a stratified analysis based on propensity score of vaccination. Vaccination status was ascertained in 333 of 598 patients with confirmed influenza and 785 of 1384 test-negative patients. Overall estimated crude vaccine effectiveness was 57% (41%, 68%). After adjusting for age, chronic comorbidities and pregnancy status, the estimated vaccine effectiveness was 37% (95% CI: 12%, 55%). In an analysis accounting for a propensity score for vaccination, the estimated vaccine effectiveness was 48.3% (95% CI: 30.0, 61.8%). Influenza vaccination was moderately protective against hospitalisation with influenza in the 2010 and 2011 seasons.

## Introduction

Influenza vaccination is required each year because of antigenic change in circulating influenza viruses and the short –term immunity induced by current haemagglutinin-based vaccines. Seasonal influenza vaccine is provided free of cost in Australia to adults aged ≥65 years, Indigenous Australian adults aged ≥15 years, those with medical comorbidities and pregnant women [1].

Although the aim of the influenza vaccination program is to prevent serious morbidity and mortality, most clinical trials have been performed in the community, where influenza is mostly a mild, self-limiting illness [2]–[4]. We have previously reported evidence of effectiveness of the influenza H1N1/09-containing vaccines against hospitalisation with H1N1/09 influenza in the 2010 season in Australia [5]. However, vaccine effectiveness against all strains appeared to be attenuated by vaccine failures in a small number of patients with non-H1N1/09 influenza.

In this study, we estimate vaccine coverage in hospitalized patients and vaccine effectiveness of the seasonal influenza vaccine against hospitalisation with confirmed influenza in the 2010 and 2011 seasons.

## Methods

### Study Setting and Design

This study was based on hospital-based surveillance conducted in sentinel hospitals in Australia. In 2010, 15 hospitals based in capital or large regional centres were involved as previously described [6] and this study includes data on 1169 patients previously published based on an analysis in 2010 [5]. In 2011, the participating hospitals were The Alfred Hospital, the Royal Melbourne Hospital, Monash Medical Centre, Geelong Hospital (Victoria), Royal Adelaide Hospital (SA), The Canberra Hospital and Calvary Hospital (ACT) and the Royal Perth Hospital (WA). Prospective active surveillance was conducted for confirmed cases of influenza presenting for admission at each hospital. We performed a prospective test-negative study, a study design similar to a case control study, by also collecting data on patients who had suspected influenza but who were negative on influenza testing (“test negative controls”). The decision to test for suspected influenza was left to the discretion of the clinician.

### Cases and Controls

Cases were defined as hospitalised adult (≥18 years) patients with influenza A or influenza B confirmed by nucleic acid detection using polymerase chain reaction (PCR). Controls were defined as the next hospitalised adult patient tested for suspected influenza but found to be negative by influenza PCR, with up to two recruited where available. Patients were identified from testing logs maintained by laboratories or infection control units at each hospital.

#### Vaccination status

Influenza vaccination was defined as follows.

- In 2010, receipt of the monovalent H1N1/09 vaccine or the seasonal trivalent vaccine (containing an A/California/7/2009 (H1N1) - like strain, an A/Perth/16/2009 (H3N2) - like strain and B/Brisbane/60/2008 - like strain) in 2010 or
- In 2011, receipt of the seasonal trivalent vaccine (containing the same strains as in 2010).

This was determined from the hospital medical record and patient self-report; primary care practitioners were not contacted as this was not within the scope of our ethical approval and privacy legislation. We included the monovalent H1N1/09 vaccine in our definition as we wanted to estimate the effectiveness of the vaccination policy, and in 2010, 79% of admissions with confirmed influenza were due to H1N1/09 influenza.

#### Other definitions

Medical risk factors were the presence of any chronic diseases that qualified patients for publicly funded vaccination including cardiac disease, chronic respiratory conditions, other chronic illnesses requiring regular medical follow up or hospitalisation in the previous year, including diabetes mellitus, chronic renal failure, chronic neurological conditions and immunosuppression. We also considered other groups that qualify for publicly funded vaccine, including age ≥65 years, pregnant women and Indigenous (Aboriginal or Torres Strait Islander) Australians ≥15 years of age. We did not consider obesity as height and weight measures were poorly documented.

### Statistical Tests

Vaccine effectiveness was estimated from the odds ratio (OR) of vaccination in cases and controls as (1-OR) ×100%. Four methods were used:

1. A crude analysis based on a conditional logistic regression stratified by site and by date of testing (in two week blocks, calculated from the epidemiological week) [5], [7].
2. Simple adjustment, based on a multivariate logistic regression adjusted for potential confounders, including the presence of medical risk factors and age ≥65 years (both included in the model), pregnancy status and Indigenous status (which were only included if statistically significant).
3. A propensity scored analysis, based on a model based on clinical covariates (and potential confounders) to predict vaccination status in control patients.
4. An imputed analysis, based on a multiple imputation procedure to augment missing vaccine status. This was used in combination with the propensity scored analysis (to assess the effect of imputation); analyses 1 and 2 were performed using non-missing data (complete set) only.

The propensity score was constructed based on clinical covariates known at the time of vaccination status in control patients (method D reported by Mansson [8]). All covariates were included in this model, whether statistically associated with vaccination or otherwise. The propensity score was calculated using the logistic regression formula. Model calibration (which represents the probability that a randomly selected vaccinated patient has a higher propensity score than a randomly selected non-vaccinated patient) was assessed using the area under the receiver operator characteristic curve. Model fit was assessed by examining the proportion of patients vaccinated and unvaccinated by decile of propensity score. The adequacy of covariate balance was assessed by the mean standardized difference across deciles of propensity score, where a difference of >10% represents a significant residual imbalance [9]. Vaccine effectiveness was estimated from the odds ratio of vaccination in cases and controls, stratifying on the decile of propensity score. A Wald test was performed to test the null hypothesis that vaccine effectiveness did not vary across different age groups.

Imputation of missing vaccination status was performed by a multiple imputation procedure implemented in Stata 12. This uses logistic models for missing status and vaccination status based on clinical covariates as well as influenza diagnosis. Fifty datasets were imputed and vaccine effectiveness was estimated from the odds ratio of vaccination in cases and control after stratification on the decile of the propensity score.

Statistical tests were performed using Stata 12 (College Station, Texas).

### Ethical Statement

Ethical approval to perform surveillance and report data was obtained from Human Research Ethics Committees of all participating hospitals and at the Australian National University. Due to the use of these non-identifiable data for public health surveillance and their non-sensitive nature, written consent was not felt to be necessary by all research ethics committees. Where patients were contacted to clarify details of their medical history, the nature of the study was explained and verbal consent was obtained, and this was documented in the medical record. All study procedures, including the waiver of written consent and use of verbal consent, were approved by the following ethics committees: Australian Capital Territory Health Human Research Ethics Committee, Australian National University Human Research Ethics Committee, Hunter New England Human Research Ethics Committee, Human Research Ethics Committee for the Northern Territory Department of Health and Menzies School of Health Research, Cairns & Hinterland Health Service District Ethics Committee, Mater Health Services Human Research Ethics Committee, Royal Brisbane and Women’s Hospital Human Research Ethics Committee, Royal Adelaide Hospital Research Ethics Committee, Tasmania Health & Medical Human Research Ethics Committee, Alfred Hospital Ethics Committee, Barwon Health Human Research Ethics Committee, Melbourne Health Human Research Ethics Committee, Southern Health Human Research Ethics Committee, Royal Perth Hospital Human Research Ethics Committee, Western Sydney Local Health District Human Research Ethics Committee, Metro South Health Service District Human Research Ethics Committee.

## Results

During the 2010–2011 seasons, there were 598 admissions to sentinel hospitals with confirmed influenza (302 admissions at 15 sites in 2010 and 296 admissions at 8 sites in 2011). Of these, 311 patients (52%) were female and the median age was 44 years (interquartile range 27, 62 years); 132 patients (22%) were 65 years or older at admission. There were 25 Indigenous patients admitted. Of all patients with confirmed influenza, 436 patients (73%) were reported to have a chronic medical condition and 33 female patients (10%) were pregnant at the time of admission.

Vaccination status was ascertained in 333 of 598 patients (56%) with confirmed influenza; a higher proportion of patients in 2010 had vaccination status ascertained (67%) than in 2011 (44%), largely due to a change in policy by an ethics committee at one site precluding patient contact. Vaccination status was ascertained in 785 of 1384 (57%) of test negative control patients (Table 1).

**Table 1:** Characteristics of patients.

	Control		Influenza A		Influenza B	
	Vaccine status ascertained	Vaccine status not ascertained	Vaccine status ascertained	Vaccine status not ascertained	Vaccine status ascertained	Vaccine status not ascertained
Number	785	599	300	238	33	27
Male	406 (52%)	325 (54%)	139 (46%)	115 (48%)	14 (42%)	19 (70%)
Age ≥65 years	282 (36%)	278 (46%)	52 (17%)	64 (27%)	9 (27%)	7 (26%)
Medical risk factors	631 (80%)	488 (81%)	226 (75%)	167 (70%)	23 (70%)	20 (74%)
Pregnant*	4 (1%)	9 (3%)	18 (11%)	13 (11%)	0 (0%)	2 (25%)
Indigenous	70 (9%)	45 (8%)	11 (4%)	12 (5%)	0 (0%)	2 (7%)
Nursing home resident	24 (3%)	40 (7%)	3 (1%)	7 (3%)	1 (3%)	0 (0%)
ICU/HDU admission	205 (26%)	147 (25%)	80 (27%)	51 (21%)	4 (12%)	4 (15%)
Pneumonia	502 (64%)	339 (57%)	113 (38%)	74 (31%)	6 (18%)	4 (15%)
Received influenza immunisation	424 (54%)		99 (33%)		10 (30%)	

* expressed as proportion of female patients.

### Vaccination Coverage

In control patients where vaccination status was ascertained, 59% of 631 patients with medical comorbidities, 82% of 282 patients aged ≥65 years and 47% of 70 Indigenous Australians were vaccinated with the either the monovalent H1N1/09 and/or the 2010 seasonal vaccine in 2010, or the 2011 seasonal trivalent vaccine in 2011.

**Figure 1:**
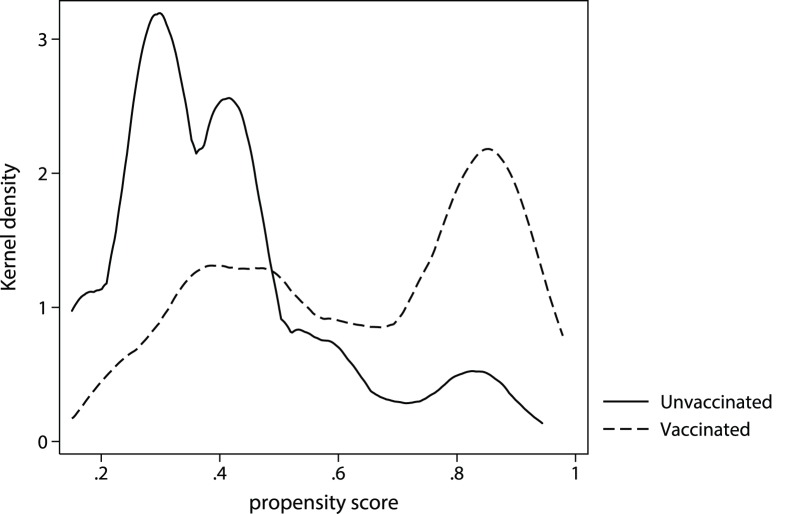
Observed vs model predicted vaccination status by decile of propensity score.

A propensity score for vaccination was constructed using all available clinical variables available to the clinician at the time of vaccination. Details of the multivariate model are listed in Table 2. The calibration of this model was moderately good (AUROC = 0.78) and the proportion of patients vaccinated increased with increasing propensity score (Figure 1). After stratifying on the decile of the propensity score, covariate balance was achieved (Table 3).

**Table 2:** Factors associated with vaccination: propensity score construction.

	Odds ratio (95% CI)
Any chronic illness	0.94 (0.53, 1.66)
Age > = 65 years	3.45 (1.50, 7.90)
Interaction age> = 65 years and chronic illness	1.69 (0.66, 4.29)
Male gender	0.93 (0.67, 1.30)
Pregnancy	2.47 (0.33, 18.73)
Indigenous ethnicity	1.37 (0.74, 2.54)
Number of medical comorbidities	1.26 (0.86, 1.84)
Chronic respiratory disease	1.59 (0.96, 2.64)
Chronic cardiac disease	1.41 (0.75, 2.66)
Current malignancy	0.72 (0.33, 1.59)
Immunosuppression	1.37 (0.75, 2.50)
Connective tissue disease	1.09 (0.36, 3.30)
Chronic neurological disease	1.06 (0.54, 2.10)
Nursing home resident	2.49 (0.71, 8.68)
Chronic renal disease	0.63 (0.31, 1.26)
Current smoker	0.48 (0.31, 0.73)

**Table 3:** Observed vaccination status and covariate balance following weighing by inverse of propensity score.

	Observed vaccinated status		Standardized differenceprior to adjustment	Standardized difference followingstratification by decile of propensity score
	Unvaccinated	Vaccinated		
Number of patients	360	424		
Age > = 65 years	52 (14.4%)	230 (54.2%)	23.0%	3.9%
Female gender	179 (49.7%)	199 (46.9%)	25.0%	1.1%
Pregnant	2 (0.6%)	2 (0.5%)	0.5%	−6.4%
indigenous	37 (10.3%)	33 (7.8%)	8.2%	5.5%
Any chronic illness	258 (71.7%)	373 (88.0%)	15.5%	−1.6%
Chronic respiratory disease	136 (37.8%)	238 (56.1%)	24.8%	−0.2%
Chronic cardiac disease	46 (12.8%)	148 (34.9%)	18.6%	−5.8%
Current malignancy	23 (6.4%)	39 (9.2%)	7.3%	7.2%
Immunosuppression	86 (23.9%)	132 (31.1%)	20.4%	6.5%
Connective tissue disease	7 (1.9%)	15 (3.5%)	2.6%	1.7%
Chronic neurological disease	28 (7.8%)	54 (12.7%)	10.1%	−4.6%
Nursing home resident	4 (1.1%)	20 (4.7%)	2.4%	11.3%
Chronic renal disease	37 (10.3%)	51 (12.0%)	10.9%	−0.7%
Current smoker	104 (28.9%)	55 (13.0%)	16.3%	−4.8%

### Vaccine Effectiveness

**Figure 2:**
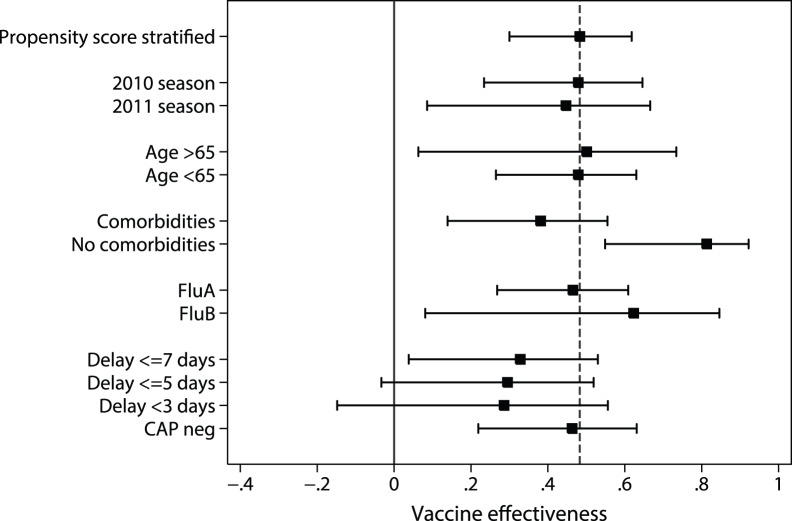
Estimated vaccine effectiveness (based on stratified analysis on propensity score) in subgroups and sensitivity analysis. Dashed line represents estimated vaccine effectiveness in all patients in primary analysis. All estimates adjusted for age group, medical comorbidities and pregnancy status.

The crude odds of vaccination in adults with confirmed influenza compared to controls was 0.43 (95% CI: 0.32, 0.59); therefore the estimated crude vaccine effectiveness was 57% (41%, 68%). After adjusting for age (≥65 years), the presence of medical comorbidities and pregnancy status (table 4), the adjusted odds of vaccination was 0.63 (0.45, 0.88); therefore, the estimated vaccine effectiveness using simple multivariate regression was 37% (95% CI: 12%, 55%). Based on an analysis stratified on the decile of the propensity score, the estimated vaccine effectiveness was 48.3% (95% CI: 30.0, 61.8%). Estimated vaccine effectiveness in subgroups is depicted in Figure 2. The estimated vaccine effectiveness in patients 50–64 years was 41% (95% CI: −2.3, 66.5%) in patients 65–80 years was 47% (95% CI: −13.6, 75.7%) and in patients >80 years was 59.2% (−27.1%, 86.9%). The Wald test did not find evidence of an interaction between the odds of vaccination in cases and controls across different age groups (p = 0.96).

**Table 4:** Factors associated with hospitalisation with confirmed influenza: simple multivariate analysis.

Factors	Crude OR (95% CI)	p	Adjusted OR (95% CI)	p
Female	1.28 (0.96, 1.70)	0.10		
Age ≥65 years	0.33 (0.23, 0.47)	<0.001	0.45 (0.31, 0.67)	<0.001
Medical comorbidities	0.69 (0.49, 0.97)	0.03	0.91 (0.63, 1.31)	0.60
Influenza vaccination	0.43 (0.32, 0.59)	<0.001	0.63 (0.45, 0.88)	0.01
Pregnancy	16.29 (4.56, 58.23)	<0.001	10.36 (2.86, 37.58)	<0.001
Indigenous	0.84 (0.37, 1.90)	0.67		
Resident in nursing home	0.53 (0.17, 1.66)	0.28		

A lower proportion of vaccinated patients with confirmed influenza were admitted to ICU compared to unvaccinated patients with confirmed influenza (20/109 (18%) vs 64/224 (29%); p = 0.045). In patients >65 years, 9 of 32 (28%) vaccinated patients were admitted to ICU compared to 5 of 20 (25%) unvaccinated patients (p = 0.5). In patients <65 years, 11 of 68 (16%) vaccinated patients and 59 of 204 (26%) unvaccinated patients were admitted to ICU (p = 0.038).

#### Imputed analysis

**Figure 3:**
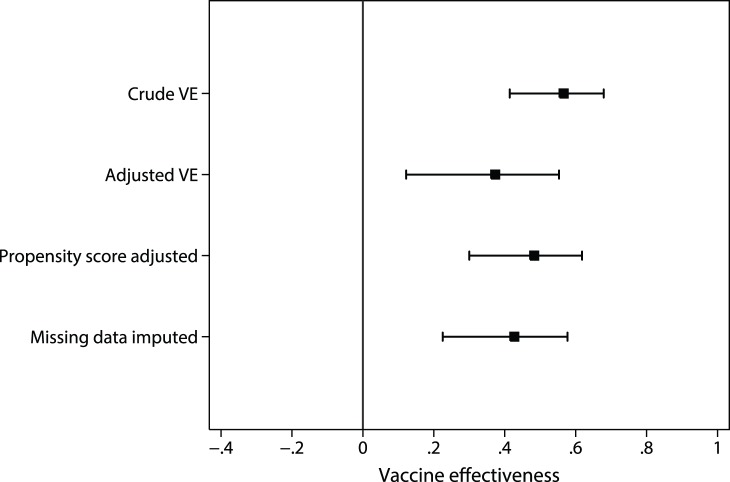
Estimated vaccine effectiveness, by method of analysis.

Details of patients in which vaccine status was not ascertained are listed in table 1. A multiple imputation procedure was performed imputing missing vaccine status based on age group, pregnancy, Indigenous ethnicity, presence of chronic respiratory disease, chronic cardiac disease, current malignancy, immunosuppression, connective tissue disease, neurological disease, nursing home residence, renal disease and influenza diagnosis. Based on an imputed set analysis and stratified on decile of propensity score, the estimated vaccine effectiveness was 42.7% (95% CI: 22.5, 57.7%). The estimated vaccine effectiveness using different methods of analysis are depicted in Figure 3.

## Discussion

Over the 2010 and 2011 seasons, there was a good match between the influenza strains in the vaccine and circulating strains [10]. Our findings suggest that vaccination is moderately protective against hospitalisation with confirmed influenza. The results were similar when either simple statistical adjustment or propensity scoring was used. In this hospitalised population, vaccination coverage was similar to that reported in national surveys.

The estimated vaccine effectiveness in this study is lower than that reported against H1N1/09 influenza in 2010 [5]. This may reflect the relative lack of genetic change in the H1N1/09 strain since it emerged in 2009, and a poorer antigenic match with other strains. We included the monovalent H1N1/09 vaccine in our definition of vaccinated in 2010, as the majority of circulating influenza was H1N1/09 strain. There are few other studies in hospitalised patients with confirmed influenza; a Spanish group found that none of 64 patients hospitalised with pandemic H1N1/09 influenza were vaccinated, compared to 9 of 101 test negative hospitalised controls [11]. A US study estimated at vaccine effectiveness of 61%, but this was based on only 39 patients with confirmed influenza [12]. A study from the Netherlands estimated only a modest effect of influenza vaccine on hospitalisation with influenza (VE 19%, 95% CI: −28%, 49%) but vaccine coverage in this population appeared to be low [13].

Most previous studies of influenza vaccination have been conducted in primary care. The vaccine effectiveness against hospitalisation from influenza may be different from that against medically presented influenza in the community for several reasons. Vaccines may be less immunogenic in the elderly, or patients with respiratory and other chronic disease may still require hospitalisation with minor infections. Several community-based studies performed since 2009 have generally shown high protective efficacy, with estimates of effectiveness of the 2010 seasonal vaccines ranging from 59%–79% [14], [15] and the monovalent H1N1/09 vaccine between 56–93%. [16]–[19].

There have been surprisingly few studies that have examined the effectiveness of influenza vaccine against serious complications. Most previous studies of influenza vaccination and hospitalisation have examined its effectiveness against hospitalisation with clinically diagnosed influenza and/or pneumonia, rather than PCR confirmed influenza [20], [21]. Although pneumonia is probably a more sensitive endpoint for influenza-related illness (as not all patients are tested for influenza, and some cases of secondary bacterial pneumonia would be PCR negative at presentation), PCR confirmed influenza is likely to be much more specific, as the majority of cases of pneumonia would be due to other pathogens against which the influenza vaccine would not be effective. Because some patients with pneumonia may have had a preceeding undiagnosed infection with influenza and might be PCR negative on admission, we performed a sensitivity analysis excluding patients with pneumonia, with similar estimates.

Studies using an endpoint of confirmed influenza have generally shown a higher effectiveness compared to those using non-specific clinical endpoints, although studies that include serological endpoints are thought to overestimate vaccine effectiveness [3]. In a recent systematic review of 17 randomized controlled trials and 14 observational studies, the pooled vaccine efficacy against confirmed influenza was estimated at 59% [3]. However, only one of these studies examined the effectiveness of influenza vaccination to prevent hospitalisation. [12].

We also found some evidence that vaccinated patients admitted with influenza were not as severely unwell as unvaccinated patients, based on a lower proportion admitted to intensive care. Some studies have found that patients with vaccine failure had milder illnesses [22] and were less likely to present to primary care practitioners [23]. This may reflect attenuation of influenza disease severity by partial protection, and appeared to be more pronounced in the non-elderly population. However, further work is required to confirm this finding in hospitalised patients and the small numbers and incomplete ascertainment of vaccination preclude further robust analysis.

The strengths of this study were that we used the highly specific outcome measure of PCR-confirmed influenza and were able to verify comorbidities from the medical record and/or patient interview. However, this study had several limitations. Immunisation status could not be ascertained in a substantial proportion of cases and controls. Excluding patients where vaccination status was not known may result in bias if the characteristics of patients where vaccination status was not known are systematically different to those where vaccination status was ascertained but only if these differences are not accounted for in an adjusted analysis. In this study, the characteristics of patients where vaccination status was not known were similar to those included in the analysis, which makes bias less likely.

We adjusted for several potential confounders, including age, the presence of medical comorbidities, and pregnancy; because publicly funded vaccine is available to these groups at risk of severe influenza, this resulted in a lower adjusted estimate of vaccine effectiveness. However, we cannot exclude the possibility of unmeasured confounding, most notably attendance at primary care, although the degree to which this is a confounder for hospitalisation with influenza is uncertain. We found older age to be protective, consistent with previous reports suggesting that this population may have been protected by prior exposure [24]. As many hospitals had discontinued the use of H1N1/09 specific PCRs in favour of assays that only distinguished between influenza A and influenza B, we were not able to provide estimates of VE against specific subtypes in 2011. However, data from national surveillance systems suggests that the majority of influenza A strains in 2010 were H1N1/09 with a substantial minority due to H3N2 subtype [10].

In the test negative study design, it is assumed that the proportion of influenza-negative patients admitted reflects the vaccination status of the general population at risk of hospitalisation, as influenza vaccination is not expected to have any effect on non-influenza respiratory illnesses. We found that the proportion of control patients ≥65 years who were vaccinated was 82% and those with comorbidities was 59%. This is similar to that reported in national surveys of vaccine coverage where 74.6% of adults ≥65 years and 53.4% of people with chronic disease were vaccinated [25]. Although we did not find the elderly and those with comorbidities to be at risk of hospitalisation with influenza, this is only in comparison to hospitalised influenza-negative patients. As the decision whether to test patients for influenza was left to the discretion of clinicians, and data are not available on patients with influenza-like illness who were not tested, we cannot exclude the possibility of selection bias. However, this would only be expected to bias the result if the decision to test was correlated with vaccination.

Both clinical trials and more recent observational data provide strong evidence that influenza vaccination is effective in reducing illness due to influenza [3]. In the two influenza seasons following the emergence of the H1N1/09 influenza, we have also found that the available influenza vaccines reduce the risk of hospitalisation in vaccinated patients compared to unvaccinated controls. This study supports public health policy to reduce severe influenza disease by immunising high-risk patients with influenza vaccine.
